# Expression Analysis of Molecular Chaperones Hsp70 and Hsp90 on Development and Metabolism of Different Organs and Testis in Cattle (Cattle–yak and Yak)

**DOI:** 10.3390/metabo12111114

**Published:** 2022-11-15

**Authors:** Yan Cui, Penggang Liu, Sijiu Yu, Junfeng He, Seth Y. Afedo, Shengnan Zou, Qian Zhang, Jun Liu, Liangli Song, Yuanfang Xu, Ting Wang, Hui Li

**Affiliations:** 1 College of Veterinary Medicine, Gansu Agricultural University, Lanzhou 730070, China; 2 College of Veterinary Medicine, Yangzhou University, Yangzhou 225009, China

**Keywords:** Hsp70 and Hsp90, expression, different organs, metabolism, cattle

## Abstract

Hsp70 and Hsp90 play an important role in testis development and spermatogenesis regulation, but the exact connection between Hsp70 and Hsp90 and metabolic stress in cattle is unclear. Here, we focused on the male cattle–yak and yak, investigated the expression and localization of Hsp70 and Hsp90 in their tissues, and explored the influence of these factors on development and metabolism. In our study, a total of 54 cattle (24 cattle–yaks and 30 yaks; aged 1 day to 10 years) were examined. The Hsp90 mRNA of the cattle–yak was first cloned and compared with that of the yak, and variation in the amino acid sequence was found, which led to differences in protein spatial structure. Using real-time quantitative PCR (RT-qPCR) and Western blot (WB) techniques, we investigated whether the expression of Hsp70 and Hsp90 mRNA and protein are different in the cattle–yak and yak. We found a disparity in Hsp70 and Hsp90 mRNA and protein expression in different non-reproductive organs and in testicular tissues at different stages of development, while high expression was observed in the testes of both juveniles and adults. Moreover, it was intriguing to observe that Hsp70 expression was significantly high in the yak, whereas Hsp90 was high in the cattle–yak (*p* < 0.01). We also examined the location of Hsp70 and Hsp90 in the testis by immunohistochemical (IHC) and immunofluorescence (IF) techniques, and the results showed that Hsp70 and Hsp90 were positive in the epithelial cells, spermatogenic cells, and mesenchymal cells. In summary, our study proved that Hsp70 and Hsp90 expressions were different in different tissues (kidney, heart, cerebellum, liver, lung, spleen, and testis), and Hsp90 expression was high in the testis of the cattle–yak, suggesting that dysplasia of the cattle–yak may correlate with an over-metabolism of Hsp90.

## 1. Introduction

Yaks (Bos grunniens) are very important for local herdsmen on the Qinghai–Tibetan Plateau of China. The cattle–yak is a hybrid that exhibits obvious heterosis of yaks and cattle, such as a tall variety, fast growth rate, drought tolerance and disease resistance [1]. Our previous study confirmed that Hsp60 proteins are expressed in different tissues (kidney, cerebellum, liver, heart, spleen and lung), and the highest expression level is in the testis of the cattle–yak. Hsp27 is located in the spermatogenic cells and mesenchymal cells of yak testicles [2,3]. It has been suggested that the possible mechanism of heat stress could cause damage to semen quality in that heat stress reduces the sensitivity of testicular cells by up-regulating HSPs [4]. The aim of this study was to investigate whether there are differences in the expression of Hsp70 and Hsp90 in various organs of the cattle–yak and yak.

Recent studies have shown that Hsp70 changes and modifies polypeptide molecules to prevent the improper folding of proteins. Hsp90 exists in the cytoplasm or endoplasmic reticulum in the form of open chains, and it can inhibit or promote the synthesis of proteins [5]. Weng et al. showed that HspA2 expression levels observably differed in various organs of the male yak, especially in the testes, followed by other organs, and its weakest expression was seen in the spleen [6]. Inducing drug inhibitions of Hsp70 and Hsp90 expression in the serum of mice can abrogate skeletal muscle catabolism and prevent the loss of muscle function as well as muscle and epididymal fat mass [7]. The increased expression of Hsp70 and Hsp90 in hyperthyroid rats’ heart tissue can protect the heart from oxidative damage and cardiovascular derangements [8]. In addition, Hsp70 and Hsp90 mRNAs are the most abundant in the fat body, followed by integument and hemocyte, and lower transcription levels are present in the Malpighian tubules and silk glands of Bombyx mori [9].

Moreover, it has been shown that Hsp70 and Hsp90 play important roles in tissue specificity in female yaks including promoting lactating function [10]. The testis is an important place for sperm cell proliferation and development. Previous studies showed that oxidative stress, Hsp70 expression, and germ cell apoptosis were decreased in MOLE-treated groups [11]. However, many factors are responsible for infertility in the male cattle–yak. These factors include testicular dysplasia, spermatogenesis disorder, an abnormal metamorphosis phase, and impaired ability of Sertoli cells to assist in germ cell differentiation in the testicular seminiferous tubule [12]. In Sertoli cells, energy metabolism is important because disorders of energy metabolism eventually result in infertility [13]. Thus, we determined the extent of Hsp70 and Hsp90 regulation of testicular development and sperm production. Cattle–yak infertility is not only due to histological differences; the regulation of genes and proteins generated by sperm are other influencing factors. Previously, studies showed that the gene is expressed in steroidogenic tissues of the testis, and using locus-specific primers, only transcripts of CYP17A1a were detected in the testis of a bovine [14]. MTHFR proteins were found to be highly localized in the cells of the seminiferous tubules of the yak testis compared to that of the cattle–yak [15], whereas the metabolite changes were associated with an increase in the percentage of seminiferous tubules with round spermatids as well as dose-dependent dead cells [16].

Focusing on HSPs, we found that they are reported to play active roles in biological processes, such as sperm movement, energy metabolism, protein processing, and oxidative stress [4]. Hsp70 plays an important role in sperm meiosis, sperm maturation, and even sperm–egg recognition. In human sperm, the expression of HspA2 in the elongated sperm and sperm cells is significantly higher than that of the primary spermatocyte [17]. Aging is associated with increased testicular levels of heat shock protein beta-1 (Hsp27) and antioxidant enzymes. On the other hand, exercise does not protect against age-induced testicular atrophy and leads to deleterious effects on sperm morphology [18]. Sudden changes in temperature during the animal breeding stage can cause Hsp60, Hsp70 and Hsp90 to rapidly respond and become highly expressed to improve the ability to protect the gap-associated protein from disturbance [19]. Nevertheless, the interconnectivity of male sterility and Hsp70 and Hsp90 of mules and cattle–yaks is still unclear. Therefore, this experimental research will provide a theoretical basis for future studies on animal hybrid sterility.

## 2. Materials and Methods

### 2.1. Experimental Animals

Organs were sampled from pasture cattle on the Tibetan plateau in Qinghai, China [20]. The animal study was reviewed and approved by the Animal Ethics Committee of Gansu Agricultural University (SYXK (Gan) 2019–0003). Written informed consent was obtained from the owners for the participation of their animals in this study. A total of 54 healthy cattle (24 cattle–yaks and 30 yaks) were included in the study (Table 1). All cattle–yaks and yaks were kept under the same natural environment (altitude, approximately 2300~2500 m; temperature, 3~6 °C; and oxygen content, 14.97%). According to the feeding habits of cattle of different ages, different methods of normal feeding were adopted. Male cattle–yaks without feeding value for herdsmen were excluded, so no sample from the senile group was included. Cattle–yaks and yaks with conventional diseases were considered healthy individuals.

### 2.2. Preparation of mRNA and Proteins

All cattle were considered clinically healthy on the basis of the results of a health assessment and disease detection. Each cattle was euthanized with sodium pentobarbital (200 mg/kg, IV). The tissue samples were obtained immediately after euthanasia. A total of 21 organs (lung, cerebellum, kidney, liver, heart, spleen and testis) were dissected, collected in a frozen liquid nitrogen cylinder, and later stored at −80 °C. Total RNA from cattle organs were isolated using TRIzol kit (Thermo Fisher Scientific, Waltham, MA, USA), and reverse-transcription polymerase chain reaction (RT-qPCR) was carried out in order to clone the cDNA.

Cryopreserved samples were ground in liquid nitrogen and transferred to centrifuge tubes together with lysis buffer RIPA/PMSF (Solarbio, Beijing, China) at appropriate volumes. After fully blending until a pale pink color was obtained, the sample tubes were incubated in ice boxes for 2 h (200 r/h). After centrifugation at 4 °C for 10 min (12,000 r/h), a complete divided protein was collected. In addition, total protein concentration for each sample was measured by microplate reader. The total protein concentration was then adjusted to the same level, 4× sample buffer was added, and the mixture was incubated at 100 °C for 12 min to completely denature the proteins.

### 2.3. Molecular Cloning of Hsp90

The reference gene sequence of Hsp90 was checked according to the National Center for Biotechnology Information (NCBI) database (Accession No. NM-001012670.2). Primers for cloning the initial codon of Hsp90 mRNA were designed according to predicted conserved region sequences in other Bos cattle animals (Table 2). The target segments were inserted into a cloning vector pMD-18T and transfected into Escherichia coli JM109 competent cells. Primers for 5′ Hsp90 and 3′ Hsp90 were designed using sequencing data. Then, the first-strand cDNA was used for Hsp90 gene cloning and sequencing.

### 2.4. Physical and Chemical Properties Analysis of Hsp90

The physical and chemical properties of Hsp90 in the cattle–yak were determined. An open reading frame in the mRNA sequence of Hsp90 was identified using NCBI (https://www.ncbi.nlm.nih.gov/nuccore/KF690731.1, accessed on 1 October 2014), and then the nucleotide was translated into amino acids using Vector NTI 11 software [19].

Homology of gene and protein sequences were performed using BLASTn and BLASTp in NCBI. The Conserved Domain (CD) search service was used for the predicted gene sequences (http://www.ncbi.nlm.nih.gov/Structure/cdd/cdd.shtml, accessed on 15 October 2014). The 3-D structure of the predicted protein was constructed according to methods described on the websites (http://bioinf.cs.ucl.ac.uk/psipred, accessed on 15 October 2014), (https://swissmodel.expasy.org/interactive, accessed on 20 October 2014), (https://web.expasy.org/protparam, accessed on 20 October 2014). The deduced nucleotide sequence of Hsp90 was aligned using Clcbio main workbench software (http://www.clcbio.com, 25 October 2014). A phylogenetic tree was constructed using Clcbio main workbench software through the neighbor-joining method, using amino acid sequences of Hsp90 from SwissProt databank/Genbank.

### 2.5. Expression of Hsp70 and Hsp90 Gene in Different Non-Reproductive Organs

The expression of Hsp70 and Hsp90 were detected in non-reproductive organs from non-reproductive systems (kidney, heart, cerebellum, lung, liver, and spleen) of cattle–yaks and yaks. The expression levels of Hsp70 and Hsp90 mRNA in different organs were detected using quantitative real-time PCR (Invitrogen, Waltham, MA, USA) with Hsp70- and Hsp90-specific primers. Beta (β)-actin was used as a control gene to normalize the expression quality of each cDNA, since it is constitutively expressed in different tissues [20].

### 2.6. Protein Expression of Hsp70 and Hsp90 in Testicular Tissues

Protein samples were thawed and mixed using a spin column (Bio-Rad), and then separated on a 5% SDS-PAGE gel for Western blot analysis. After gel electrophoresis, proteins were transferred to nitrocellulose (NC) membranes (Millipore Corporation, Billerica, MA, USA). The membranes containing protein were blocked with 5% fat-free milk in TBST at room temperature (18~24 °C) for 2 h, and then hybridized using anti-Hsp70-and-Hsp90 antibody (1:1000, Abcam, Hong Kong) or rabbit polyclonal antibody (1:2000, Abcam, Hong Kong) at 4 °C overnight. The membrane was then washed four times with TBST and labeled with HRP-conjugated secondary antibody (1:4000,) for 2 h at room temperature (18~24 °C). After washing five times with 1× TBST, Hsp70 and Hsp90 were detected on the membrane with an ECL detection kit (Beyotime, Shanghai, China). The protein expression intensity was determined by optical density analysis. The intensities of β-actin bands were used for the single standard [20].

### 2.7. Positive Reaction Analysis of Hsp70 and Hsp90 Protein

All organs and testicular tissues from the cattle were fixed in 4% paraformaldehyde solution at room temperature (18~24 °C) for hebdomad. The tissue was cut into pieces (0.5~1 mm^3^ each), paraffin-embedded, and the sections (4 µm) were sliced, dried on glass sides, and stored.

Experimental Samples were dewaxed using dimethyl benzene and then dehydrated with an increasing alcohol gradient for immunohistochemical staining to investigate the Hsp70- and Hsp90-positive reaction. Endogenous peroxidase was eliminated with 3% deionized H_2_O_2_ (18~22 min), and sections were hydrochloric-acid-rehydrated and sealed with goat serum (18~22 min). Afterwards, they were incubated overnight at 4 °C with primary rabbit anti-Hsp70 and mouse anti-Hsp90 monoclonal antibodies (1:300, Abcam, Hong Kong, China). Then, the primary antibody was washed with PBS and the sections were incubated with secondary antibody. Then, the labeled samples were counterstained with 3,3′-diaminobenzidine to show the nuclei. In addition, immunofluorescence should be stained with fluorescence-labeled secondary antibodies, thus re-dyeing the nuclei was not necessary.

### 2.8. Measurement and Statistical Analyses

The results of RT-qPCR were analyzed by variance. The intensity in the Western blot analyses and the immunohistochemical and immunofluorescence assays were measured using integrated optical density analysis and Image-Pro plus 6.0. All measured data were calibrated according to standards and analyzed with SPSS 21.0. The data ratio basis was determined between β-actin and sample protein levels. Gene and protein expression levels were analyzed by one-way ANOVA and Duncan’s post hoc test. *p*-values less than 0.01 were considered highly statistically significant, and values less than 0.05 were considered statistically significant [3].

## 3. Results

### 3.1. Analysis of Physical and Chemical Properties of Hsp90

The cDNA sequence of Hsp90 was cloned and submitted to GenBank with accession number KF690731.

**Figure 1:**
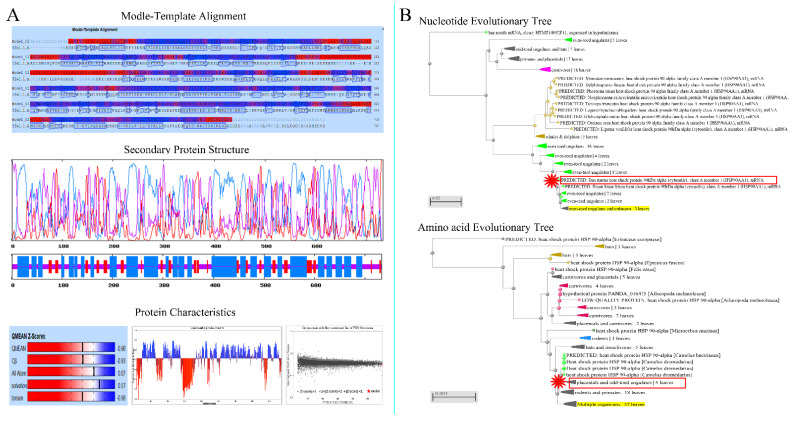
Physicochemical and evolutionary tree analysis of Hsp90 in cattle–yak. (**A**) The physical and chemical properties of Hsp90 in cattle–yak. Modle-template alignment, secondary protein structure and protein characteristics. (**B**) Homology analysis of amino acids and nucleotides.

The nucleotide and amino acid sequences of Hsp90 were analyzed. The Hsp90 nucleic acids were 3064 bp long. The results from the contig analysis showed that the predicted Hsp90 cDNA of 3064 bp contained an ORF of 2202 bp from 146 bp to 2347 bp. Physical and chemical properties and the spatial structure of amino acids are shown in the attached table (Figure S1). To verify the results, three primers, Hsp90-1, Hsp90-2, and Hsp90-3, were used to clone the full-length ORF of Hsp90. The RT-PCR results showed that the isolation of the 2202 bp cDNA fragment from the cattle–yak total RNA (heart sample) was successful, and the amino acid composition was analyzed (Table S1, Figure 1). Finally, the confirmed cDNA sequence was deposited in the GenBank database with accession number KF690731. To obtain genomic DNA of Hsp90, the publicly available cow genome database at NCBI (http://www.ncbi.nlm.nih.gov/projects/genome/guide/cow, accessed on 25 October 2021) was screened using the Hsp90 cDNA sequence as a query. A cow (Bos taurus) contig (GenBank Accession No. NM_001012670) encompassing the entire Hsp90 nucleotide sequence was identified by BLASTGen analysis.

The atomic number of the protein-coding region was 11710. Hsp90 had a molecular formula of C_3733_H_5950_N_986_O_1202_S_27_, molecular weight of 847408.84 Da, and theoretical isoelectric point of 4.841. Hsp90 had a half-life of approximately 30 h, instability index of 42.77, fat-soluble index of 79.80, and average hydrophobicity index of −0.744 (Table S2).

**Figure 2:**
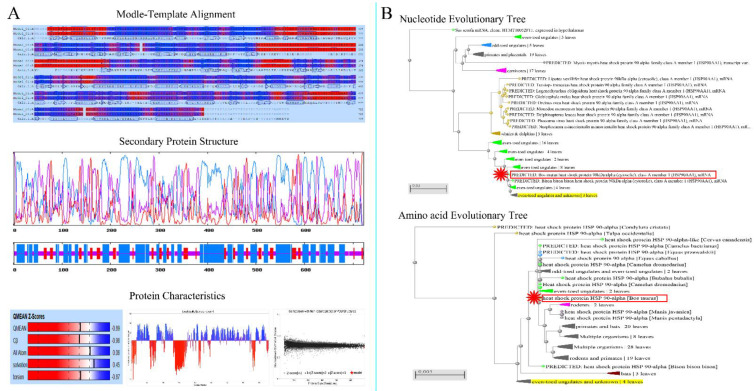
Physicochemical and evolutionary tree analysis of Hsp90 in yak. (**A**) The physical and chemical properties of Hsp90 in yak. Modle-template alignment, secondary protein structure and protein characteristics. (**B**) Homology analysis of amino acids and nucleotides.

The evolutionary tree was constructed to analyze the species homology of Hsp90 nucleotide sequences (Figure 2). The analysis of the nucleotide homology tree showed that the cattle–yak Hsp90 evolutionarily shared a higher sequence identity with even-toed ungulates, such as the *bison, B. taurus, B. grunniens, Mustela putorius furo, Capra hircus, Ailuropoda melanoleuca, Sorex araneus, Cavia porcellus, and Equus caballus*.

### 3.2. Analysis of Structure Specificity of Hsp90 Protein

The amino acid sequence of Hsp90 was aligned with known *B. grunniens* sequences through BLASTp. The result indicated that Hsp90 proteins were different from other members in the HSPs family. The Hsp90 homology ratio was approximately 99.56%, 99.33%, 96.91%, 87.83%, and 86.90% identical to those of HSPs from *B. grunniens, B. taurus, C. hircus, E. caballus, and Mus musculus*, respectively (Figure 1B and Figure 2B).

**Figure 3:**
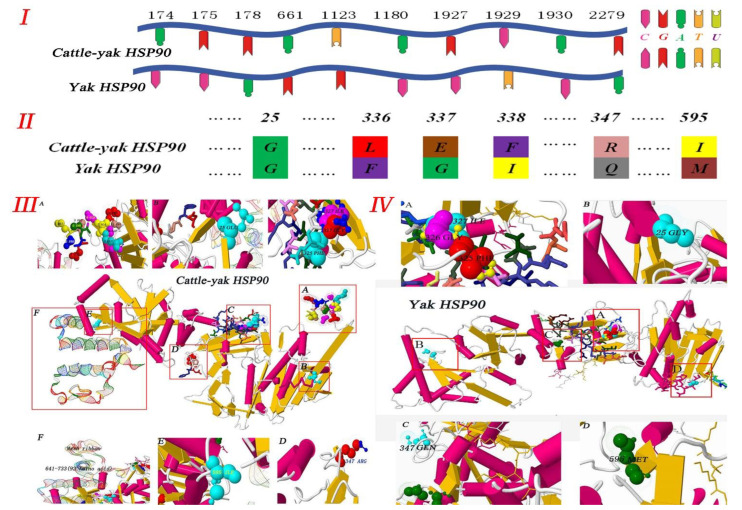
Analysis of Hsp90 protein structure in cattle–yak and yak. (**I**) and (**II**) Hsp90 nucleotide and amino acid sequence analysis showing the sites and types of mutations in cattle–yak and yak. (**III**) Hsp90 protein structure of cattle–yak. (A) The initiation terminal in cattle–yak is much more than in yak and the following amino acids are shown: MET, PRO, GLU, GLU, THR, GLN, ALA, GLN, ASP, and PRO, PRO. (B), (D), (E), (F) Mutation sites and amino acids are as follows: 25 (GLU), 36 (THR), 37 (PHE), 38 (TYR), 347 (ARG), 595 (ILE). (C) More than 92 amino acids are at the terminal. (**IV**) Hsp90 protein structure in yak. (A), (B), (C), (D) Mutation sites and amino acids are as follows: 14 (GLY), 25 (THR), 26 (PHE), 27 (TYR), 336 (GLN), 584 (MET).

The cattle–yak Hsp90 protein sequence showed that the mutation of nucleotides caused changes in the amino acid sequence, such as changing F-G-I residues into L-E-F residues (Figure 3). Most importantly, there was a higher spatial structure of the protein H-key number (397), spiral number (30), link number (23), and corner number (89), and more details in Figure S2 and Table S3. In addition, compared with the yak, the initiating terminal and end terminal of the cattle–yak were longer.

### 3.3. Expression and Distribution of Hsp70 and Hsp90 in Non-Reproductive Organs

**Figure 4:**
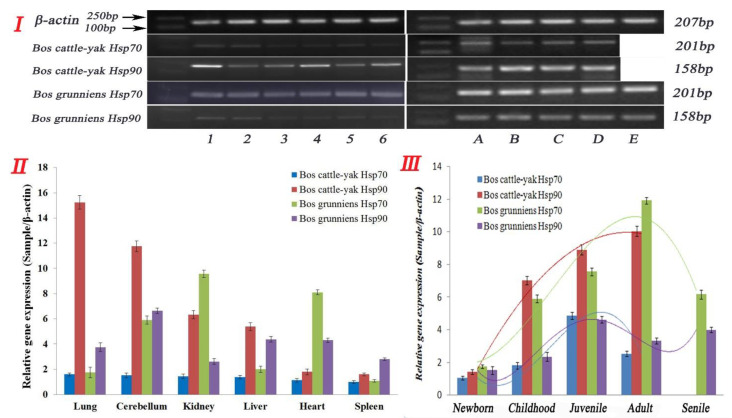
Gene expression in different tissues of cattle–yak and yak. (**I**) The results of RT-PCR. Lanes: (1) lung; (2) cerebellum; (3) kidney; (4) liver; (5) heart; and (6) spleen. Lanes: (A) newborn; (B) childhood; (C) juvenile; (D) adult; and (E) senile. (**II**) The gene expression levels of Hsp70 and Hsp90 in non-reproductive tissues from cattle–yak and yak. (**III**) Gene expression levels of Hsp70 and Hsp90 in testicular tissues from male cattle (cattle–yak and yak). Note: The curved lines represent expression trends of Hsp70 and Hsp90 proteins in different organs and different developmental stages in testis. Different colors represent different expressions of Hsp70 and Hsp90 in the organs and testicular tissue from cattle–yak and yak.

Hsp70 and Hsp90 mRNA expression in the tissues of the cattle–yak and yak were investigated through real-time RT-PCR analysis (Figure 4I), using the total RNA isolated from cattle–yak and yak organs as templates. Each sample was repeated three times for statistical analysis. As shown in Figure 4II, we examined six organs in the cattle–yak and yak and Hsp70 and Hsp90 was widely expressed in all organs (n = 6, adult cattle, 3–6 years). According to our data, Hsp70 and Hsp90 were both reduced in the cattle–yak compared to the yak, and the trend of reduced expression was from the lung, cerebellum, kidney, liver, heart to the spleen (n = 6, *p* < 0.01). There was no significant difference in Hsp70 expression within these organs, but intriguingly, the expression levels of Hsp90 were consistently higher than those of Hsp70 in almost all the organs tested (n = 6, *p* < 0.01). In the meantime, although the expressions of Hsp70 and Hsp90 were both higher in the cerebellum, kidney, and heart in the yak, the expressions of Hsp70 and Hsp90 in different tissues and levels were irregular. Taken together, our data suggest that the expression of Hsp90 was consistently higher than that of Hsp70 in all the organs tested (n = 6, *p* < 0.01) in the cattle–yak, but irregular in the yak (Table S4).

**Figure 5:**
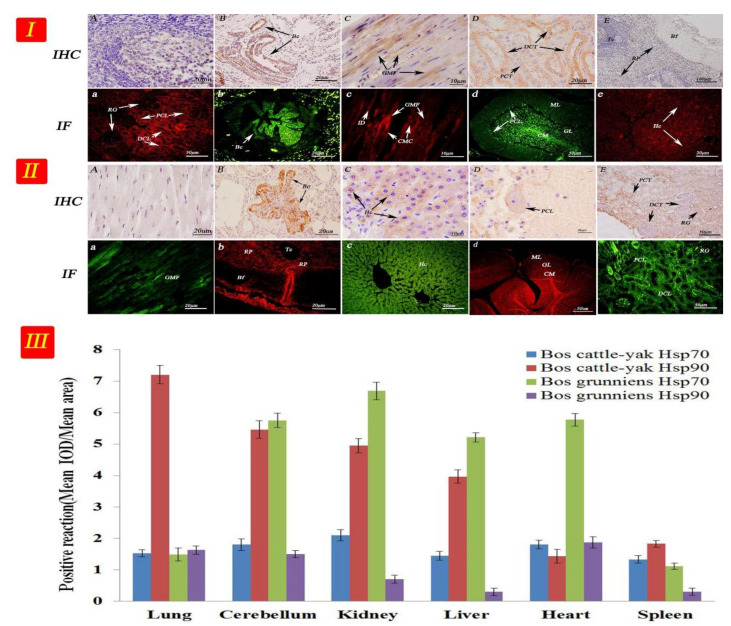
Hsp70 and Hsp90 expression of in diffrent tissues. The arrow represents the site of positive expression. IHC(A,B,C,D,E) and IF(a,b,c,d,e). (**I**) Cattle-yak: (A) Control section from yak spleen, without immunoreactions (negative control). (B), (D) Positive staining for Hsp90 was observed in the terminal bronchioles, distal convoluted tubule, and proximal convoluted tubule of lungs and kidney. (C), (E) Positive staining for Hsp70 was observed in the cardiac muscle fibers and red pulp of heart and spleen. (a), (c), (e) Positive staining for Hsp70 was observed in the distal convoluted tubule, proximal convoluted tubule, cardiac muscle fibers and hepatocytes in kidney, heart, and liver. (b), (d) Positive staining for Hsp90 was observed in the terminal bronchioles, cerebellar medulla, granular layer and Purkinje cell layer of lungs and cerebellum. (**II**) Yak: (A) Control section from yak heart, without immunoreactions (negative control). (B), (D) Positive staining for Hsp70 was observed in the terminal bronchioles, cerebellar medulla, granular layer and Purkinje cell layer of lungs and cerebellum. (C), (E) Positive staining for Hsp90 was observed in hepatocytes, distal convoluted tubule, and proximal convoluted tubule of liver and kidney. (a), (c), (e) Positive staining for Hsp90 was observed in the cardiac muscle fibers, hepatocytes, distal convoluted tubule, and proximal convoluted tubule of heart, liver, and kidney. (b), (d) Positive staining for Hsp70 was observed in the red pulp, cerebellar medulla, granular layer, and Purkinje cell layer of the spleen and cerebellum. Note: Terminal bronchiole (TB); hepatocyte (Hc); central vein (CV); cardiac muscle fibers (CMF); distal convoluted tubule (DCT); Purkinje cell layer (PCL); renal glomerulus (RG); biofilm (Bf); trabecula (Tc); red pulp (RP); white pulp (WP); cerebellar medulla (CM); molecular layer (ML); granular layer (GL); and proximal convoluted tubule (PCT). (**III**) The result of optical density analysis value. The curved lines represent expression trends of HSP proteins in different organs, and different color represents different proteins.

Hsp70 and Hsp90 expression was mainly observed in the cardiac muscle cells, kidney tubules, hepatocytes, cerebellar medulla, and Purkinje cells (Figure 5I,II). Positioning analysis indicated that the Hsp70 and Hsp90 proteins were mainly concentrated in the reticular tissue and epithelia. Thus, the proteins were mainly deposited in the cell membrane and in the cytoplasm but not the nucleus. Furthermore, Hsp90 protein expression levels were markedly lower than those of Hsp70 in almost all the tissues tested (n = 6, *p* < 0.01), except in the lung (Figure 5III and Table S4).

### 3.4. Expression and Distribution of Hsp70 and Hsp90 in Testicular Tissues at Different Developmental Stages

There were differential expressions of Hsp70 and Hsp90 between the cattle–yak and yak in six non-reproductive organs. We further examined Hsp70 and Hsp90 expression in the testis of cattle–yak and yak at different stages of development. As shown in Figure 4III, Hsp70 and Hsp90 gene expression levels were significantly different in the testes of the cattle–yak and yak at different developmental stages (n = 6, *p* < 0.01). In the cattle–yak, Hsp70 expression showed an obvious trend from newborn to adult, increasing to highest at the juvenile stage and then decreasing at the adult stage with low expression in senile animals; meanwhile, Hsp90 expression kept increasing in the adult cattle–yak (n = 6, *p* < 0.01). It is worthy to note that the expression of Hsp90 was significantly higher than that of Hsp70 in every developmental stage of the testis, whereas in the yak, Hsp70 expression was highest in the adult testis. Hsp90 expression was highest in the juvenile testis and showed a great difference in the cattle–yak, in which Hsp70 expression was also higher during every developmental stage of the testis (n = 6, *p* < 0.01) (Figure 4III).

**Figure 6:**
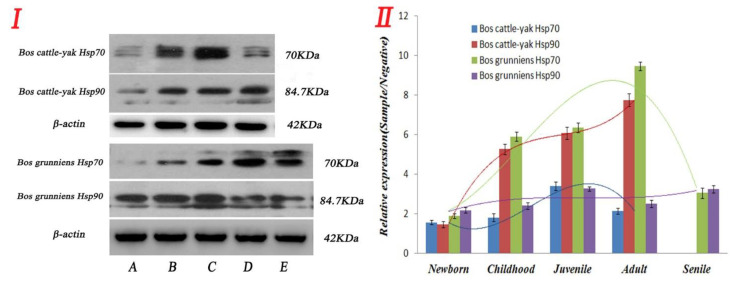
Hsp70 and Hsp90 expression in testicular tissue of cattle–yak and yak. (**I**) Schematic diagram of labeled bands and their sizes. (**II**) Different colors represent expressions of Hsp70 and Hsp90 in different testicular tissues from cattle–yak and yak.

**Figure 7:**
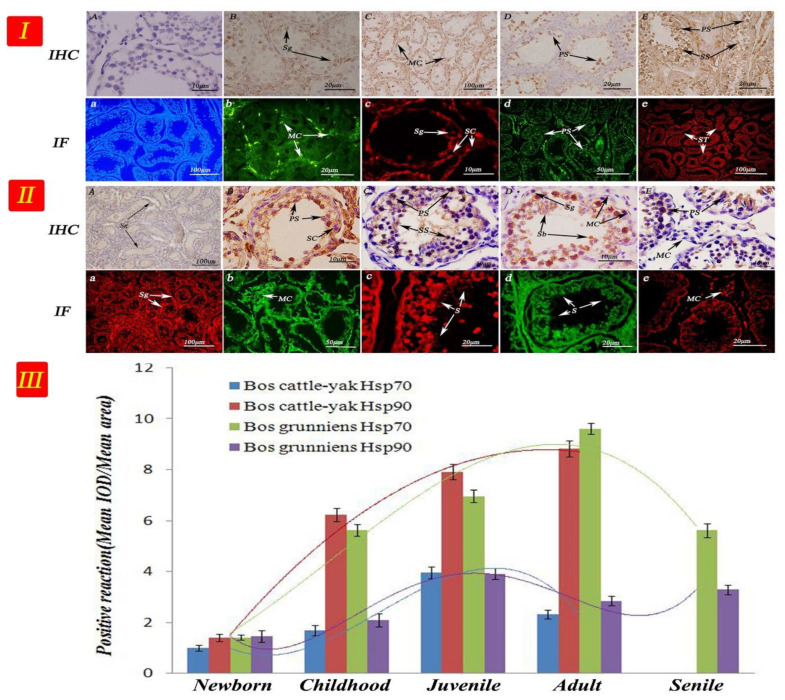
IHC and IF expression of Hsp70 and Hsp90 in testicular tissues from cattle–yak and yak. The arrow represents the site of positive expression. IHC(A,B,C,D,E) and IF(a,b,c,d,e). (**I**) Cattle-yak: (A) Control section from testicular tissue of adult yak, without immunoreactions (negative control). (B), (D) Positive staining for Hsp70 was observed in spermatogonium and primary spermatocyte of newborn and juvenile testis. (C), (E) Positive staining for Hsp90 was observed in mesenchymal, primary spermatocyte, and secondary spermatocyte cells of testes from childhood and adulthood. (a) Control section from testicular tissue of adult yak, without immunoreactions (negative control). (b), (d) Positive staining for Hsp90 was observed in the mesenchymal cells and primary spermatocyte of newborn and adult testis. (c), (e) Positive staining for Hsp70 was observed in spermatogonium, Sertoli cells, and seminiferous tubule of testes from childhood and adulthood. (**II**) Yak: (A), (C), (E) Positive staining for Hsp90 was observed in spermatogonium, secondary spermatocyte, and mesenchymal cells of newborn, juvenile, and senile testis. (B), (D) Positive staining for Hsp70 was observed in primary spermatocyte, Sertoli cells, spermoblast, and spermatogonium of testes from childhood and adulthood. (a), (c), (e) Positive staining for Hsp70 was observed in the spermatogonium, sperm and mesenchymal cells of newborn, juvenile, and senile testis. (b), (d) Positive staining for Hsp90 was observed in the mesenchymal cells and sperm of testes from childhood and adulthood. Note: Primary spermatocyte (PS); secondary spermatocyte (SS); spermatogonium (Sg); spermoblast (Sb); sperm (S); Sertoli cells (SC); mesenchymal cells (MC); myoid cells (MC); seminiferous tubule (ST). (**III**) The results of optical density analysis value. The curved lines represent expression trends of HSP proteins in different testicular tissues, and different colors represent different proteins.

As shown in Figure 6, the protein expression levels of Hsp70 and Hsp90 were detected in testicular tissues at different development stages in the cattle–yak and yak. The results showed that the Hsp90 protein expression level was significantly higher than that of Hsp70 in almost all developmental stages of the cattle–yak (n = 6, *p* < 0.01). In contrast, the Hsp70 protein expression level was significantly higher than that of Hsp90 in almost all developmental stages of the yak (n = 6, *p* < 0.01), except the newborn stage (Figure 6II and Figure 7III and Table S5).

The seminiferous tubules of newborn cattle were thin and sparse (Figure 7I,II). As cattle–yaks aged, the number of spermatogenic cells at different developmental stages decreased, in addition few sperm and sperm cells appeared. Hsp90 was highly expressed in the spermatocyte in cattle–yak testicular tissue, whereas the expression levels in the myogenic cells and basement membrane were the weakest. In contrast, the Hsp70 expression levels in the spermatocyte were the weakest. We were interested in the expressions that were strong, as such, as the organism developed, luminals enlarged, seminiferous tubules became closely packed, and different amounts of sperm cells appeared during the development of sperm. For the elderly yak, the amount of spermatogenic cells in the contorted seminiferous tubules decreased significantly. The Hsp90 expression was the strongest in yak testis in the primary spermatocyte, followed by that in the secondary spermatocyte. Sperm cells also showed Hsp90 expression, contrary to spermatogonium cells. The Hsp70 expression levels in mesenchymal cells were strong, while expression levels in the basement membrane and myogenic cells were the weakest.

## 4. Discussion

For the first time, we isolated, sequenced, and characterized cDNA clones that encode Hsp90 from the cattle–yak. Research has found that differences in the protein spatial structure leads to Hsp90 functional differences [21,22]. Using our reference sequence, our analytical results showed that three ORFs in the Hsp90 sequence illustrated the frame-shifting property of the sequence. We also presented evidence that similarities between cattle–yak Hsp90 and that of yak, B. taurus, and goats were very high, as shown in the phylogenetic tree (Figure 1 and Figure S1), and that the cattle–yak evolution together with that of the above-mentioned animals have very close homology. In eukaryotic cells, HSP gene promoters upstream of TATA have a heat shock element with a size of approximately 20 bp, which is necessary in the transcription of specific gene sequences (C-GAA-TTC-G) [23,24]. The study revealed different expression levels for both proteins and long non-coding RNAs and showed that the function and metabolic pathways of differently expressed proteins were related to reproductive processes [25]. Our results showed (Figure 3) that cattle–yak amino acids have a multipoint mutation relative to the yak, and we evaluated the loss of genetic diversity caused by hatchery selection or internal control gene in the cattle–yak. Based on the above findings, we believed that amino acid mutation is an important factor of the Hsp90 functional differences.

The Hsp70 and Hsp90 gene and protein expression levels were significant not only in different species but also in different organs of the same animals. Interestingly, comparing HSP expression in males and females, the females had significantly lower post-exercise Hsp72 levels that were not influenced by estradiol supplementation. This characteristic is mainly displayed in the heart, liver, lung, and in the red and white vastus muscle [26]. In addition, HSPs under cold stress induce oxidative stress in the spleen, which influences immune function in chicks. Hsp70- and Hsp90-organizing mRNA and protein levels were significantly higher in gastric cancer tissues than in normal tissues in human blood [27,28]. With these data from different viewpoints, the appropriate physiological function of different tissues could also be verified. We found that the Hsp70 gene and protein expression was higher in the kidney, heart, and cerebellum of cattle. However, Hsp90 had a higher expression in the lung, cerebellum, and liver of cattle. Studies have shown that apoptosis reduced when Hsp90 expression was inhibited [21]. At the same time, proper dietary boron treatment might accelerate ostrich spleen development by promoting Hsp70 expression and inhibiting apoptosis, while a high amount of boron supplementation could impair ostrich spleen structural development by inhibiting the Hsp70 expression level and promoting cell apoptosis [29]. An increase in Hsp70 and Hsp90 was described in plasma and mononuclear cells and various organs and tissues, such as muscle, liver, cardiac tissue, and brain, after an acute bout of exercise [30]. Considering the high expression in the cerebellum and heart of the cattle–yak and yak, we speculated that Hsp70 and Hsp90 expression could protect cells and tissues from natural environmental factors.

Different stimuli could induce HSPs in the proximal convoluted tubule of the lung with significant differential expression in the bronchioles and respiratory bronchiole epithelial cells and smooth muscle cells [31]. According to findings, the continuous exposure to thiram caused the abnormal function of myocardial tissues. Meanwhile, the expression of heat shock proteins (Hsp60, Hsp70, Hsp90) markedly decreased in the thiram-treated groups [32]. The Hsp70 and Hsp90 gene and protein expression levels were significantly up-regulated when porcine fetal fibroblast and oviduct epithelial cells were stimulated by the cold [33]. Studies have shown that increased HSPs protect chicken testicular tissues from damage caused by inflammation and, in this case, the gene and protein levels of Hsp70, Hsp60 and Hsp90 were up-regulated [34]. Researchers showed that the metabolic surgery to improve insulin resistance caused the normalization of circulating levels and liver concentrations of Hsp70 and Hsp90 [35]. In glycolytic oxidative stress of porcine skeletal muscle, Zearalenone decreased the protein abundance of Hsp27 and pHsp27, while heat stress increased the protein expression of Hsp70 and Hsp90 [36]. This study demonstrated the localization of Hsp70 and Hsp90 in the cell membrane and cytoplasm, but not the nuclei of cells of organs. It is an undeniable fact that Hsp70 and Hsp90 have an active functions in tissue physiology in the cattle–yak and yak.

**Figure 8:**
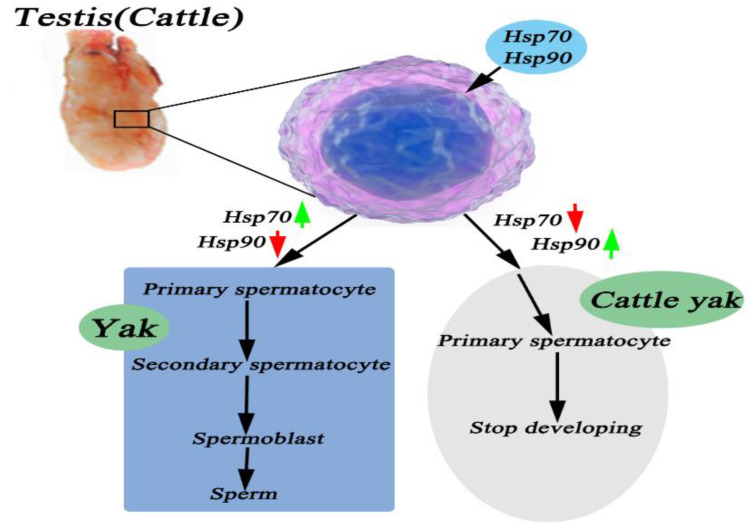
Hsp70 and Hsp90 schematic diagram of action mechanism in testicular tissues. The highly expressed Hsp90 could block development of primary spermatocyte in testis of cattle–yak. The highly expressed Hsp70 and decreased Hsp90 could promote sustained development from spermatocyte to mature sperm in testis of yak.

Transcriptome results indicated that the overexpression and knockdown of Pgam1 in Sertoli cells resulted in the up-regulation of 458 genes (117 down-regulated, 341 up-regulated) and the down-regulation of 409 genes (110 down-regulated, 299 up-regulated) [37]. We examined which expression in terms of the Hsp70 and Hsp90 gene and protein was altered in cattle–yak to elucidate the mechanism by which Hsp70 and Hsp90 affected the development of testicular tissues. Encouragingly, Hsp90 expression was generally higher than Hsp70 in cattle–yak testicular tissues, especially during childhood, the juvenile stage, and adulthood. Spinaci et al. reported that the Hsp90 expression level gradually reduced when pig sperm was frozen and the protein content reduced before sperm quality declined [38]. Meanwhile, the expression in cattle–yak remained at higher levels in the juvenile and adult. This result is consistent with results obtained by Abd El-Fatta, who showed that high expression of Hsp90 could inhibit testicular dysfunction caused by DEHP [39]. In the infertile male cattle–yak, the expression of was HSFY2 delayed, not absent. There is therapeutic potential of leucine for oxidative testicular injury, as evidenced by its ability to attenuate oxidative stress and proinflammation while stalling cholinergic dysfunction and modulating nucleotide hydrolysis, as well as for modulating oxidative dysregulated metabolites and their pathways [40,41]. In addition, heat stress caused spermatogenesis and semen quality damage by reducing the sensitivity of testicular cells to androgen through Hsp70 up-regulation. Lower Hsp70 and Hsp90 expressions led to low sperm quality of the boar [4,42]. Hsp70 expression may have been up-regulated as a protective mode against apoptosis in the spermatozoa of infertile men [43]. Thus, as in Figure 8, we speculated that the high expression of Hsp90 in testicular tissues of male cattle activate the restoration of normal fertility in the male cattle–yak.

We analyzed the Hsp70 and Hsp90 expression in testicular tissues at different stages of yak development to establish whether the Hsp70 and Hsp90 content has a positive correlation with the reproductive capacity of the yak. The results indicated that the highest Hsp70-2 gene expression level was in the yak testicular tissue, followed by that of the cattle and cattle–yak. In addition, it was reported that Hsp70 protein expression was negligible at six months in the treatment group, but its intensity increased in spermatids after eleven months of treatment, similar to the control group [44]. This result is largely consistent with our experimental result in that the Hsp70 expression efficiency in yak testicular tissue gradually increased with age and metabolism, and the strongest expression was observed during the adult stage, then it gradually decreased in old age. In the hematological tests of Sahiwal and Kankrej breeds, the molecular response was driven by a significant up-regulation of all the key HSPs—Hsp70, Hsp90, Hsp60, and Hsp40, except Hsp27—during the hotter months of summer and hot, humid seasons [45]. Xue et al. reported that the spermatogenesis-related genes, which include Spag6, Spag16, Sox5, Sox6, and Sox13, apoptosis-related genes of the mitochondria, such as the CytC and Bcl-2, and a mutant Hsp70-2 could inhibit the coupling of a nuclear chromosome entering meiosis [46,47]. Furthermore, in the rabbit testis, Hsp90 expression levels increased first and then decreased later during the testicular development process [48]. From Figure 8, we speculated that a low expression of Hsp90 and high expression of Hsp70 were activated during normal reproduction in yak.

The immune response of the HSP family of proteins is mainly concentrated in the epithelial tissue and serous cells. Cedenho et al. found that Hsp70-2 was expressed in normal testis and testicular tissues with disturbance in sperm maturation [49]. Economou et al. reported on the developmental stage and cell-specific expression as well as the intracellular localization of the small Hsp27 during oogenesis and spermatogenesis in the Mediterranean fruit fly [50]. In our study, the expression was strongest in yak testis in the primary spermatocyte and attenuated in the secondary spermatocyte. Sperm cells also showed expression, but contrary to spermatogonium cells. Thus, we demonstrated that the low metabolism of Hsp70 correlated with male cattle–yak sterility. Hsp90 was highly expressed in the spermatocyte in cattle–yak testicular tissue, especially in mesenchymal cells. In 2003, Cardozo et al. declared with evidence that there was an increase in immune response intensity in testicular tissue with disturbances in sperm production, and the most obvious effect was in the myoid cells [51]. Detected in the frozen semen of buffaloes in different seasons, the levels of Hsp70 were significantly higher in the summer season compared to the winter season [52]. In addition, a lack of sperm causes infertility and hastens spermatocyte apoptosis and metabolism in spermatogenic epithelium. Even more remarkably, the mule, a classic example of a hybrid sterile mammal, also exhibits a similar spermatogenesis breakdown and failure at the first meiotic stage [53]. Thus, the results showed that a high expression of Hsp90 caused the abnormal metabolism of spermatocyte development. In this study, we showed an important breakthrough in the research of male metabolic infertility using the cattle–yak.

## 5. Conclusions

A change in amino acid sequence was the main cause of functional differences in the Hsp90 protein. Hsp70 and Hsp90 exhibited obvious differential metabolic expressions in different organ tissues and in different ages of cattle–yak and yak testes. In addition, Hsp70 and Hsp90 were mainly involved in metabolism in the epithelium of non-reproductive organs, spermatogenic cells, and mesenchymal cells in testicular tissues. At the core of our study, Hsp70 and Hsp90 showed specific expressions in yak and cattle–yak testicular tissues, and importantly, this correlated with cattle–yak metabolic infertility.

## Figures and Tables

**Table 1:** Source and quantity of experimental animals.

Number Cattle–yak + Yaks	Group	Age	Source	Application
6 + 6	Newborn	1~7 days	Xi’ning City Qinghai Province	Molecular biology Histology Measurement
6 + 6	Calf	5~6 months		
6 + 6	Juvenile	1~1.5 years		
6 + 6	Adult	3~6 years		
0 + 6	Senior	8~10 years		

**Table 2:** Primer sequences of target and house-keeping genes used in the study.

Primer	Sequence(5′→3′)	Tm (°C)	Note
Name			
Hsp90-1R	GATGAATACTCTGCGAACATACAA	56.9	RT-PCR
Hsp90-1F	ATGCCCGAGGAGACCCA	58.6	1113bp
Hsp90-2R	TGTTTGCTGTCCAGCCGTAT	58.9	RT-PCR
Hsp90-2F	GGAGGAGCGGAGAATAAAGG	58.2	1238bp
Hsp90-3R	CCCGATGTATGGACAATGACTC	59.2	RT-PCR
Hsp90-3F	GAAAGTTGAAAAGGTGGTTGTG	56.8	979bp
β-actin-F	GACCCAGATCATGTTTGAGACC	58.0	RT-PCR
β-actin-R	ATCTCCTTCTGCATCCTGTCAG	58.0	598bp
Hsp70-R	GCCTTGGTCTCCCCTTTGTAG	58.0	RT-qPCR
Hsp70-F	GCTGAACCCGCAGAACACG	58.0	158bp
Hsp90-R	GCTGAATAAAACCCGACACCA	62.0	RT-qPCR
Hsp90-F	CAAGCAAGATCGAACCCTCAC	62.0	174bp
β-actin-R	GCTCGGCTGTGGTGGTAAA	59.0	RT-qPCR
β-actin-F	AGGCTGTGCTGTCCCTGTATG	59.0	207bp

## Data Availability

The data presented in this study are available in the main article and the Supplementary Materials.

## Supplementary Materials

The following supporting information can be downloaded at: https://www.mdpi.com/article/10.3390/metabo12111114/s1, Figure S1: Physical and biochemical analysis of nucleotides; Figure S2: Amino acid spatial structure simulation. Table S1: Amino acid composition; Table S2: Low-level structural properties of amino acids; Table S3: Advanced structural properties of amino acids; Table S4: RT-qPCR results of Hsp70 and Hsp90 gene in different tissues and organs of yak; Table S5: Integrated optical density of Hsp70/90 expression in different tissues and organs of yak.
