# Supplementary material for: Expression Analysis of Molecular Chaperones Hsp70 and Hsp90 on Development and Metabolism of Different Organs and Testis in Cattle (Cattle–yak and Yak)
Source: Metabolites. 2022 Nov 15;12(11):1114. doi: 10.3390/metabo12111114 (PMC9694778; doi:10.3390/metabo12111114)
Supplement: Supplementary file 1 [file metabolites-12-01114-s001.zip › Table S1.pdf]

Table S1. Amino acid composition

	Bos grunniens		Bos cattle-yak	
	Quantity	Percentage(%)	Quantity	Percentage(%)
Ala (A)	33	4.6%	34	4.6%
Arg (R)	29	4.0%	30	4.1%
Asn (N)	31	4.3%	31	4.2%
Asp (D)	54	7.5%	55	7.5%
Cys (C)	7	1.0%	7	1.0%
Gln (Q)	23	3.2%	24	3.3%
Glu (E)	93	12.9%	97	13.2%
Gly (G)	34	4.7%	32	4.4%
His (H)	12	1.7%	12	1.6%
Ile (I)	51	7.1%	51	7.0%
Leu (L)	61	8.4%	62	8.5%
Lys (K)	80	11.1%	80	10.9%
Met (M)	20	2.8%	20	2.7%
Phe (F)	25	3.5%	25	3.4%
Pro (P)	19	2.6%	22	3.0%
Ser (S)	43	6.0%	43	5.9%
Thr (T)	40	5.5%	41	5.6%
Trp (W)	4	0.6%	4	0.5%
Tyr (Y)	25	3.5%	25	3.4%
Val (V)	38	5.3%	38	5.2%
Pyl (O)	0	0.0%	0	0.0%
Sec (U)	0	0.0%	0	0.0%
