# Supplementary material for: Expression Analysis of Molecular Chaperones Hsp70 and Hsp90 on Development and Metabolism of Different Organs and Testis in Cattle (Cattle–yak and Yak)
Source: Metabolites. 2022 Nov 15;12(11):1114. doi: 10.3390/metabo12111114 (PMC9694778; doi:10.3390/metabo12111114)
Supplement: Supplementary file 1 [file metabolites-12-01114-s001.zip › Table S2.pdf]

Table S2 Low-level structural properties of amino acids

	Bos grunniens	Bos cattle-yak
Number of amino acids	722	733
Molecular weight	83363.36	84741.81
Theoretical pI	4.98	4.92
Carbon (C)	3674	3733
Hydrogen (H)	5859	5950
Nitrogen (N)	971	986
Oxygen (O)	1179	1202
Sulfur (S)	27	27
Formula	$C_{3674}H_{5859}N_{971}O_{1179}S_{27}$	$C_{3733}H_{5950}N_{986}O_{1202}S_{27}$
Total number of atoms	11710	11898
Instability index	41.12	42.77
Aliphatic index	80.33	79.80
Grand average of hydropathicity (GRAVY)	-0.722	-0.744
