# Supplementary material for: Expression Analysis of Molecular Chaperones Hsp70 and Hsp90 on Development and Metabolism of Different Organs and Testis in Cattle (Cattle–yak and Yak)
Source: Metabolites. 2022 Nov 15;12(11):1114. doi: 10.3390/metabo12111114 (PMC9694778; doi:10.3390/metabo12111114)
Supplement: Supplementary file 1 [file metabolites-12-01114-s001.zip › Table S3.pdf]

Table S3. Advanced structural properties of amino acids

	Bos grunniens	Bos cattle-yak
Biounit Oligo State	Hetero-tetramer	Hetero-tetramer
QSQE	0.74	0.73
Method	EM, 9.00A	EM, 9.00A
Seq Similarity	0.57	0.57
Coverage	0.99	0.98
Range	4-688	15-699
Alpha helix (Hh)	358 (49.58%)	373 (50.89%)
Extended strand (Ee)	105 (14.54%)	102(13.92%)
Random coil	259 (35.87%)	258 (35.20)
