# Supplementary material for: Expression Analysis of Molecular Chaperones Hsp70 and Hsp90 on Development and Metabolism of Different Organs and Testis in Cattle (Cattle–yak and Yak)
Source: Metabolites. 2022 Nov 15;12(11):1114. doi: 10.3390/metabo12111114 (PMC9694778; doi:10.3390/metabo12111114)
Supplement: Supplementary file 1 [file metabolites-12-01114-s001.zip › Table S4.pdf]

Table S4 RT-qPCR results of *HSP70/90* gene in different tissues and organs of yak

**Tissues and Organs**	**ΔCT**	**ΔΔCT（ΔCT $_{sample}$-ΔCT $_{ovary/oviduct}$）**	**$2^{-ΔΔCT}$**
**Bos cattle-yak Hsp70**			
Lung	1.5944±0.1081	0.5944±0.1081	1.509845
Cerebellum	1.5183±0.1841	0.5183±0.1841	1.432267
Kidney	1.4486±0.1786	0.4486±0.1786	1.364715
Liver	1.3823±0.1407	0.3823±0.1407	1.303418
Heart	1.1485±0.1382	0.1485±0.1382	1.108416
**Spleen**	1.0000±0.1211	0.0000±0.1211	1
Newborn	1.0667±0.1141	0.0667±0.1141	1.047318
Calf	1.8066±0.1931	0.8066±0.1931	1.749085
Juvenile	2.5367±0.2186	1.5367±0.2186	2.901301
Adult	4.8500±0.1607	3.8500±0.1607	14.42001
**Bos cattle-yak Hsp90**			
Lung	15.245±0.5327	14.245±0.5327	19416.56
Cerebellum	11.755±0.4246	10.755±0.4246	1728.135
Kidney	6.3207±0.3292	5.3207±0.3292	39.96596
Liver	5.3855±0.3096	4.3855±0.3096	20.901
Heart	1.7991±0.2236	0.7991±0.2236	1.740015
Spleen	1.5981±0.1105	0.5981±0.1105	1.513722
Newborn	1.4218±0.1435	0.4218±0.1435	1.339598
Calf	7.0267±0.2567	6.0267±0.2567	65.19548
Juvenile	8.8937±0.3107	7.8937±0.3107	237.8157
Adult	10.040±0.3219	9.040±0.3219	526.3943
**Bos grunniens Hsp70**			
Lung	1.7467±0.4024	0.7467±0.4024	1.67795
Cerebellum	5.8933±0.3310	4.8933±0.3310	29.71872
Kidney	9.5800±0.3012	8.5800±0.3012	382.6814
Liver	1.9933±0.2431	0.9933±0.2431	1.990733
Heart	8.0967±0.1982	7.0967±0.1982	136.8736
Spleen	1.0900±0.0989	0.0900±0.0989	1.06437
Newborn	1.7639±0.1127	0.7639±0.1127	1.698075
Calf	5.9000±0.2335	4.9000±0.2335	29.85706
Juvenile	7.5552±0.2472	6.5552±0.2472	94.03983
Adult	11.910±0.2127	10.910±0.2127	1924.143
Senile	6.1646±0.2648	5.1646±0.2648	35.86737
**Bos grunniens Hsp90**			

Lung	3.7467±0.3387	2.7467±0.3387	6.711801
Cerebellum	6.6367±0.2136	5.6367±0.2136	49.7526
Kidney	2.6100±0.2222	1.6100±0.2222	3.052518
Liver	4.3800±0.2206	3.3800±0.2206	10.41073
Heart	4.3000±0.1785	3.3000±0.1785	9.849155
Spleen	2.8033±0.1083	1.8033±0.1083	3.490177
Newborn	1.5247±0.2346	0.5247±0.2346	1.438634
Calf	2.3500±0.2657	1.3500±0.2657	2.549121
Juvenile	4.6135±0.2094	3.6135±0.2094	12.23973
Adult	5.3212±0.1882	4.3212±0.1882	19.98991
Senile	3.9971±0.1775	2.9971±0.1775	7.983934
