# Supplementary material for: Expression Analysis of Molecular Chaperones Hsp70 and Hsp90 on Development and Metabolism of Different Organs and Testis in Cattle (Cattle–yak and Yak)
Source: Metabolites. 2022 Nov 15;12(11):1114. doi: 10.3390/metabo12111114 (PMC9694778; doi:10.3390/metabo12111114)
Supplement: Supplementary file 1 [file metabolites-12-01114-s001.zip › Table S5.pdf]

Table S5 Integrated optical density of *HSP70/90* expression in different tissues and organs of yak

**Tissues and Organs**<br>**Bos cattle-yak Hsp70**	**Integral optical density**<br>（IntDen/Area）	**Relative optical density**<br>（sample/β-action）
Lung	16.8203±1.1936	1.5233±0.1081
Cerebellum	19.8756±2.0328	1.8021±0.1841
Kidney	23.1882±1.9721	2.1032±0.1786
Liver	15.9745±1.5536	1.4467±0.1407
Heart	19.9120±1.5260	1.8033±0.1382
Spleen	14.6826±1.3372	1.3297±0.1211
Newborn	17.1901±1.2599	1.5568±0.1141
Calf	19.9441±2.1322	1.8062±0.1931
Juvenile	23.6299±2.4138	2.1401±0.2186
Adult	37.4599±1.7744	3.3925±0.1607
**Bos cattle-yak Hsp90**		
Lung	79.5411±3.2319	7.2035±0.2927
Cerebellum	60.2893±3.0321	5.4601±0.2746
Kidney	54.5839±2.5308	4.9433±0.2292
Liver	43.8179±2.3034	3.9683±0.2086
Heart	15.7901±2.4689	1.4302±0.2236
Spleen	20.1472±1.2201	1.8246±0.1105
Newborn	16.1224±1.5845	1.4601±0.1435
Calf	58.0389±2.8345	5.2562±0.2567
Juvenile	67.1851±3.4307	6.0845±0.3107
Adult	85.4993±3.5544	7.7431±0.3219
**Bos grunniens Hsp70**		
Lung	16.3797±2.2349	1.4834±0.2024
Cerebellum	63.5325±2.5507	5.7537±0.2310
Kidney	73.8677±3.0150	6.6897±0.2812
Liver	57.5156±1.5801	5.2088±0.1431
Heart	63.7201±2.1885	5.7707±0.1982
Spleen	12.3218±1.0921	1.1159±0.0989
Newborn	20.8826±1.2941	1.8912±0.1172
Calf	65.1632±2.5783	5.9014±0.2335
Juvenile	59.0714±2.7296	5.3497±0.2472
Adult	104.592±2.3486	9.4722±0.2127
Senile	33.6107±2.9239	3.0439±0.2648
**Bos grunniens Hsp90**		
Lung	17.9499±1.5315	1.6256±0.1387
Cerebellum	16.5552±1.2544	1.4993±0.1136
Kidney	7.7106±1.3493	0.6983±0.1222
Liver	3.2849±1.3317	0.2975±0.1206
Heart	20.6519±1.9709	1.8703±0.1785
Spleen	3.3005±1.1958	0.2989±0.1083
Newborn	24.0759±1.4863	2.1804±0.1346

Calf	26.5439±1.8297	2.4039±0.1657
Juvenile	36.03115±1.2079	3.2631±0.1094
Adult	38.8005±2.0781	3.5139±0.1882
Senile	35.84894±1.9599	3.2466±0.1775
