# Supplementary figures and images for: Expression Analysis of Molecular Chaperones Hsp70 and Hsp90 on Development and Metabolism of Different Organs and Testis in Cattle (Cattle–yak and Yak)
Source: Metabolites. 2022 Nov 15;12(11):1114. doi: 10.3390/metabo12111114 (PMC9694778; doi:10.3390/metabo12111114)

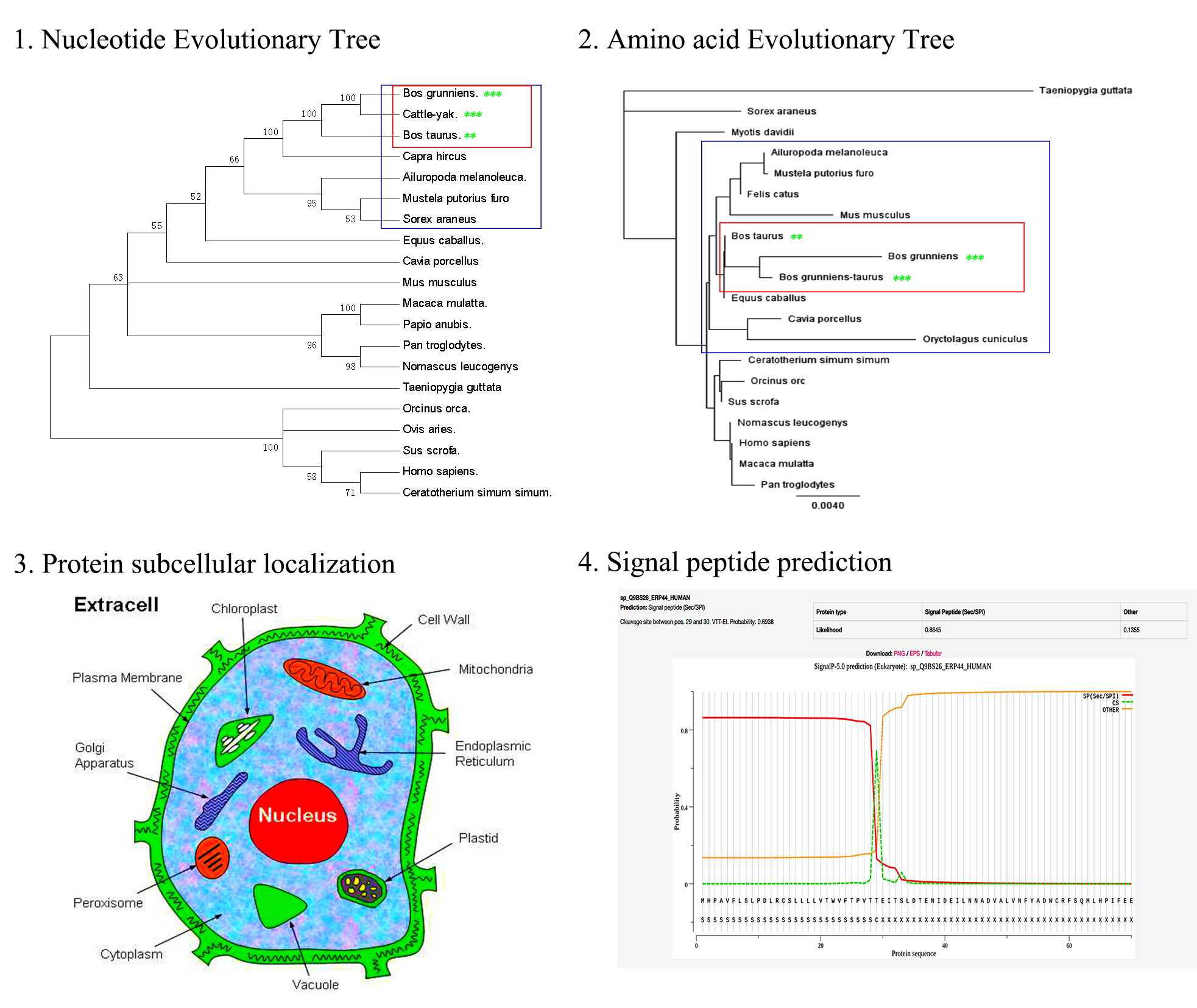

Supplement: Supplementary file 1 [file metabolites-12-01114-s001.zip › Figure S1.tif]

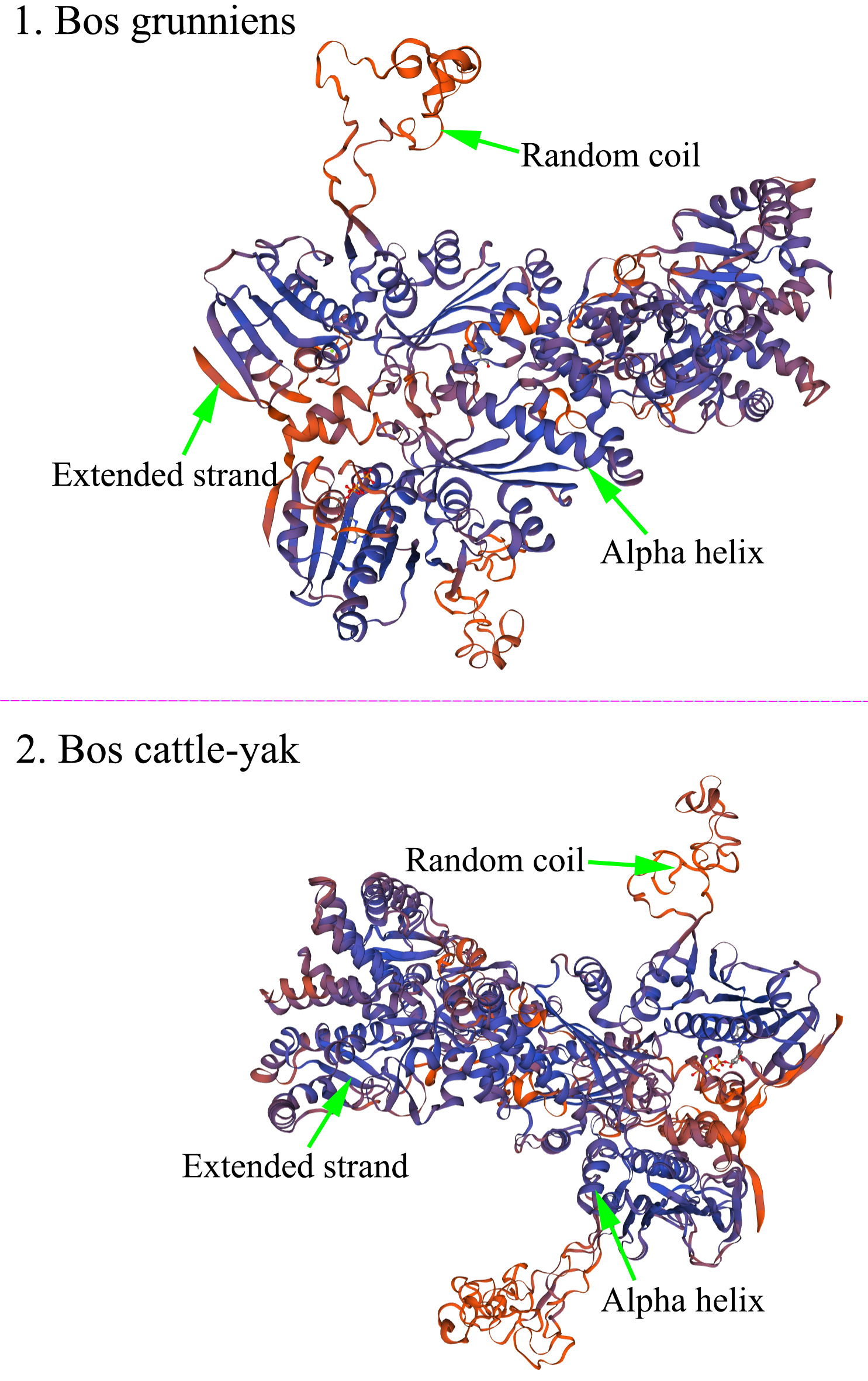

Supplement: Supplementary file 1 [file metabolites-12-01114-s001.zip › Figure S2.tif]
